# Lower pulmonary vein-to-left atrium volume ratio predicts poor rhythm outcome after atrial fibrillation catheter ablation

**DOI:** 10.3389/fcvm.2022.934168

**Published:** 2022-07-15

**Authors:** Jae-Hyuk Lee, Inseok Hwang, Hee Tae Yu, Tae-Hoon Kim, Jae-Sun Uhm, Boyoung Joung, Moon-Hyoung Lee, Hui-Nam Pak

**Affiliations:** ^1^Department of Cardiology, Myongji Hospital, Hanyang University Medical Center, Gyeonggi-do, South Korea; ^2^Department of Internal Medicine, Division of Cardiology, Yonsei University College of Medicine, Yonsei University Health System, Seoul, South Korea

**Keywords:** atrial fibrillation, catheter ablation, pulmonary vein, left atrium, computed tomography

## Abstract

Although left atrial (LA) dimension (LAD) is one of the predictors of atrial fibrillation (AF) recurrence after catheter ablation, repetitive recurrences occur in patients without enlarged LAD. We explored the predictive value of pulmonary vein (PV) to LA volume percent ratio (PV/LA%vol) for rhythm outcomes after AF catheter ablation (AFCA). We included 2913 patients (73.5% male, 60.0 [52.0–67.0] years old, 60.6% paroxysmal AF) who underwent AFCA. We evaluated the association between PV/LA%vol and AF recurrence after AFCA and compared the predictive value for AF recurrences according to the LA size with LAD. We additionally investigated the association between PV/LA%vol and *PITX2* gene using a genome-wide association study. LAD affected 1-year recurrence only in the highest tertile group (T3, *p* = 0.046), but PV/LA%vol determined 1-year recurrence in all LAD groups (T1, *p* = 0.044; T2, *p* = 0.021; and T3, *p* = 0.045). During 20.0 (8.0–45.0) months of follow-up, AF recurrence rate was significantly higher in patients with lower PV/LA%vol (Log-rank *p* = 0.004, HR 0.91 [0.84–1.00], *p* = 0.044). In the T1 and T2 LAD groups, predicting AF recurrences was better with PV/LA%vol than with LAD (AUC 0.63 vs. 0.51, *p* < 0.001 at T1; AUC 0.61 vs. 0.50, *p* = 0.007 at T2). We replicated *PITX2*-related rs12646447, which was independently associated with PV/LA%vol (β = 0.15 [0–0.30], *p* = 0.047). In conclusion, smaller PV volumes after LA volume adjustments have genetic background of *PITX2* gene and predictive value for poorer rhythm outcomes after AFCA, especially in patients without LA enlargement.

## Introduction

Catheter ablation is the most effective method of rhythm control for atrial fibrillation (AF). However, this progressive disease is accompanied by continuous long-term recurrence despite invasive procedures (1, 2). The left atrial (LA) dimension (LAD) measured by echocardiography is traditionally known as a reliable predictor of post-AF catheter ablation (AFCA) recurrence (3). However, repeated recurrences after AFCA still occur, even in patients without enlarged LA. Invasive or processed indicators such as LA pressure and LA voltage have predictive power for the recurrence risk after AFCA (4–6). However, there are limited risk factors that can predict post-AFCA recurrences in AF patients with normal or mildly remodeled LA. It has been well established that the pulmonary veins (PVs) play an essential role in initiating and maintaining AF (7). The relationship between the PV morphology and AF triggers was previously reported (8, 9). However, no study has examined the morphological relationship between PV-LA and AF recurrence after AFCA. In addition, AF is a heritable disease and *PITX2* is known as the most common AF-associated genome among over 100 related SNPs that has a significant effect on PV-LA development (10, 11). However, little is known about the relationship between *PITX2* genome and PV-LA morphology and AF recurrence.

Therefore, herein, we explored the risk factors for AF recurrence after AFCA in patients without significant LA enlargement by using imaging index representing the morphological relationship between PV and LA. We also compared and evaluated the correlation between the PV-LA morphological index and *PITX2* genome.

## Materials and methods

### Study population

The study protocol adhered to the principles of the Declaration of Helsinki and was approved by the Institutional Review Board of the Yonsei University Health System. All patients provided written informed consent for inclusion in the Yonsei AF Ablation Cohort. A total of 2,913 consecutive patients who underwent *de novo* AFCA between March 2009 and January 2020 in a single center were prospectively enrolled in this study. In all patients, we performed transthoracic echocardiography and cardiac computed tomography (CT) within 3 months before the procedure. We divided the patients into three groups according to the LAD tertile value and compared their clinical parameters, including the 1-year recurrence rate after AFCA. We also divided patients into three groups based on the tertile value of PV to LA volume percent ratio (PV/LA%vol) measured on three-dimensionally (3D) reconstructed CT. The exclusion criteria were as follows: (1) AF refractory to electrical cardioversion; (2) no available data on LA and PV volume measured on CT; (3) AF with rheumatic valvular disease; and (4) prior AF ablation or cardiac surgery. All patients stopped all anti-arrhythmic drugs (AAD) for a period corresponding to at least five half-lives before the AFCA.

### Contrast-enhanced cardiac computed tomography scan and measurement of pulmonary vein and left atrial volume

We acquired contrast-enhanced 64-slice cardiac multi-detector row CT (MDCT) (Somatom Sensation 64; Siemens Medical Solutions, Forchheim, Germany) scans by injecting bolus of 60–80 mL of iopamidol (Iopamiro 370; Bracco, Italy) at a flow rate of 5 mL/s. We reconstructed the CT images at the end-systolic and mid-diastolic phases, using a slice thickness of 0.75 mm. The volumes of the LA and PV were measured with 3D reconstructed images using an Advantage Workstation Volume Share 4.6 (GE Healthcare, United States). We measured the LA volume from the mitral valve area to the posterior wall of the LA, and PV volume from the PV ostia to primary tributaries with equal horizontal bilateral extension (Supplementary Figure 1). In case of common ostium of PV, PV volume was measured from common ostium to the primary bifurcation of superior and inferior PVs. After the summation of each PV volume, we calculated PV/LA%vol by dividing PV volume by LA volume and multiplying the value by 100. The correlation coefficients for the intraobserver reliability was 0.97 for the 3D-CT volumetric analysis.

### Genetic analysis for PV/LA%vol

Genomic deoxyribonucleic acid (DNA) was obtained from blood samples using the QuickGene DNA Whole Blood Kit S with a QuickGene mini 80 (KURABO, Osaka, Japan). DNA genotyping data were obtained using the Axiom Precision Medicine Research Array (PMRA; Thermo Fisher Scientific, MA, United States). We searched a PV/LA%vol-associated single nucleotide polymorphism (SNP) in *PITX2* gene, the most common AF-associated genome that affects PV development, using PMRA data. Cohorts 1 and 2 included 1,020 patients and 1,131 patients, respectively, and we investigated PV/LA%vol-associated SNP with *p*-values < 0.05. Linkage disequilibrium pruning was conducted to eliminate all relevant variants with variance inflation factor of 0.2. After that, we analyzed the genome-wide association study (GWAS) data of 2,051 patients out of 2,913 included patients. Using PV/LA%vol-associated SNP, we investigated the association between PV/LA%vol and *PITX2* among the included patients.

### Electrophysiological studies and catheter ablation

The electrophysiological mapping method and AFCA technique/strategy used during the study period were consistently performed as described previously (12). In brief, open irrigated-tip catheter (Celsius, Johnson & Johnson Inc., Diamond Bar, CA, United States; NaviStar ThermoCool, Biosense Webster Inc., Diamond Bar, CA, United States; ThermoCool SF, Biosense Webster Inc.; ThermoCool SmartTouch, Biosense Webster Inc.; Coolflex, Abbott Inc., Minnetonka, MN, United States; FlexAbility, Abbott Inc.; and TactiCath, Abbott Inc.) was used to deliver radiofrequency energy for the ablation under 3D electroanatomical mapping (NavX, Abbott, Inc., Chicago, IL, United States; CARTO system, Biosense Webster, Diamond Bar, CA, United States) merged with 3D CT. High-quality voltage maps were acquired using a circumferential mapping catheter during high right atrial pacing at 500 ms. We obtained the contact bipolar electrograms of 500–1,000 points on the LA endocardium and calculated the mean LA electrogram voltage by analyzing the peak-to-peak amplitude. All patients initially underwent a circumferential PV isolation. Depending on each patient’s condition and degree of atrial remodeling, the roof line, posterior-inferior line, anterior line, or complex fractionated atrial electrogram-guided ablation were added at the operator’s discretion. The procedure was considered complete when there was no immediate recurrence of AF after cardioversion with isoproterenol infusion. In the case of mappable AF triggers, extra-PV foci were mapped and ablated as much as possible. Systemic anticoagulation was achieved with intravenous heparin, while an activated clotting time of 350–400 s was maintained during the procedure.

### Post-ablation management and follow-up

All patients visited the scheduled outpatient clinic at 1, 3, 6, and 12 months after the AFCA and every 6 months thereafter or whenever symptoms occurred. All patients underwent electrocardiography at every visit and 24-h Holter recording at 3 and 6 months, then every 6 months for 2 years, annually at 2–5 years, and then biannually after 5 years according to the modified 2012 HRS/EHRA/ECAS expert consensus statement guidelines (13). Whenever patients reported palpitations, we obtained Holter or event monitor recordings to check for arrhythmia recurrence. AF recurrence was defined as any episode of AF or atrial tachycardia lasting at least 30 s. Any electrocardiographic documentation of AF recurrence 3 months after the blanking period was identified as clinical recurrence.

### Statistical analysis

Continuous variables are expressed as mean ± standard deviation for normally distributed variables and as median (interquartile range) for non-normally distributed variables and were compared using Student’s *t*-test and the Wilcoxon rank-sum test, respectively. We used the chi-square or Fisher’s exact test to compare categorical variables reported as counts (percentages). Three or more groups were compared using one-way analysis of variance, and the Bonferroni method was used to account for multiple comparisons between groups. Linear regression analysis was used to investigate the variables related to PV/LA%vol. We conducted a Kaplan–Meier analysis with log-rank test to analyze the probability of freedom from AF recurrence. The proportional hazard assumption was tested based on the Schoenfeld residuals. Using Cox regression analysis, we identified predictors of AF recurrence after AFCA. The variables selected for the multivariate analysis were those with a *p*-value < 0.05 on univariate analysis. Two multivariate models were separately presented because LAD and PV/LA%vol had a multicollinearity to each other. The area under the receiver operating characteristic curve (AUC) was used to investigate the predictive power of the parameters. The hazard ratios (HRs) and AUC of AF recurrence among patients who underwent circumferential PV isolation alone were additionally investigated as a sensitivity analysis. We used Statistical Package for the Social Sciences version 25.0 for Windows (IBM Corporation, Armonk, NY, United States) and R software version 3.6.2 (The R Foundation for Statistical Computing, Vienna, Austria) for the data analysis.

## Results

### Characteristics of patients with a low PV/LA%vol

We enrolled total of 2,913 patients (73.5% male, 60.0 [52.0–67.0] years, 60.6% with paroxysmal AF) who underwent *de novo* AFCA. Table 1 presents the baseline characteristics according to the LAD tertiles. Depending on the T1–T3 of LAD, there was a higher proportion of older (*p* < 0.001) men (*p* < 0.001) and non-paroxysmal AF (*p* < 0.001), longer AF duration (*p* = 0.011), and comorbidities in the higher tertile LAD groups (Table 1). In the cardiac imaging analyses, the higher tertile LAD group had lower ejection fraction (*p* < 0.001), higher ratio of the peak mitral flow velocity of the early rapid filling to the early diastolic velocity of the mitral annulus (E/Em, *p* < 0.001) and PV volume (*p* < 0.001), and lower PV/LA%vol (*p* < 0.001) (Table 1).

**TABLE 1:** Baseline characteristics among the three groups according to the tertile value of LA dimension.

	All subjects (*n* = 2,913)	LA dimension, 1st tertile (≤38 mm) (*n* = 1,003)	LA dimension, 2nd tertile (>38 mm and < 45 mm) (*n* = 1,082)	LA dimension, 3rd tertile (≥45 mm) (*n* = 828)	*P*
Age (year)	60.0 (52.0–67.0)	58.0 (49.0–65.0)^a^	60.0 (53.0–67.0)^b^	61.0 (54.0–68.0)^c^	<0.001
Male sex, n (%)	2140 (73.5)	686 (68.4)^a^	816 (75.4)	638 (77.1)^b^	<0.001
Paroxysmal AF, n (%)	1765 (60.6)	783 (78.1)^a^	682 (63.0)^b^	300 (36.2)^c^	<0.001
AF duration	20.0 (8.0–45.0)	17.0 (7.0–36.5)^a^	21.0 (8.0–45.0)	23.0 (9.0–48.0)^b^	0.011
Comorbidity, n (%)					
Hypertension	1369 (47.0)	355 (35.4)^a^	525 (48.5)^b^	489 (59.1)^c^	<0.001
Diabetes	438 (15.0)	103 (10.3)^a^	177 (16.4)^b^	158 (19.1)^c^	<0.001
Stroke/TIA	320 (11.0)	83 (8.3)^a^	129 (11.9)^b^	108 (13.0)^b^	0.002
Vascular disease	275 (9.4)	70 (7.0)^a^	101 (9.3)^b^	104 (12.6)^c^	<0.001
Heart failure	382 (13.1)	71 (7.1)^a^	128 (11.8)^b^	183 (22.1)^c^	<0.001
Body mass index (kg/m^2^)	24.7 (23.0–26.7)	23.7 (22.0–25.4)^a^	25.0 (23.5–26.9)^b^	25.7 (24.0–27.8)^c^	<0.001
CHA_2_DS_2_-VASc score	1.0 (1.0–3.0)	1.0 (0–2.0)^a^	1.0 (1.0–3.0)^b^	2.0 (1.0–3.0)^b^	<0.001
Echocardiography parameters					
LA dimension (mm)	41.0 (37.0–45.0)	35.0 (33.0–37.0)^a^	41.0 (40.0–43.0)^b^	48.0 (46.0–50.0)^c^	<0.001
LV ejection fraction (%)	64.0 (60.0–69.0)	65.0 (60.0–69.0)^a^	64.0 (60.0–69.0)^a^	63.0 (58.0–68.0)^b^	<0.001
E/Em	9.0 (7.3–12.0)	8.0 (7.0–10.2)^a^	9.1 (7.7–12.0)^b^	10.3 (8.3–13.3)^c^	<0.001
CT parameters					
LA volume (cm^3^)	147.2 (121.9–178.5)	124.2 (106.7–144.0)^a^	147.0 (126.5–169.4)^b^	184.1 (156.5–214.4)^c^	<0.001
PV volume (cm^3^)	5.3 (4.1–7.1)	4.9 (3.9–6.5)^a^	5.4 (4.2–6.8)^b^	5.9 (4.5–7.9)^c^	<0.001
PV/LA%vol (%)	3.7 (2.8–4.8)	4.0 (3.1–5.3)^a^	3.6 (2.8–4.8)^b^	3.3 (2.5–4.3)^c^	<0.001
PV anatomy, n (%)					0.631
Normal PV anatomy	2625 (90.1)	913 (91.0)	974 (90.0)	738 (89.1)	
Common PV trunk	83 (2.8)	27 (2.7)	33 (3.0)	23 (2.8)	
Accessory PV	205 (7.0)	63 (6.3)	75 (6.9)	67 (8.1)	
Ablation time (min)	63.0 (39.9–84.5)	58.1 (36.7–76.8)^a^	62.5 (38.9–82.3)^a^	72.1 (45.3–100.0)^b^	<0.001
Procedure time (min)	154.5 (111.0–191.0)	145.0 (103.0–180.0)^a^	153.5 (108.0–189.0)^a^	171.0 (129.0–213.0)^b^	<0.001
Ablation lesion, n (%)					
CPVI	2913 (100)	1003 (100)	1082 (100)	828 (100)	1
Empirical extra-PV LA ablation	694 (23.8)	134 (13.4)^a^	214 (19.8)^b^	346 (41.8)^c^	<0.001
Mean LA voltage (mV) (*n* = 1,918)	1.4 (0.9–1.9)	1.5 (1.1–1.9)^a^	1.4 (0.9–2.0)^a^	1.2 (0.7–1.7)^b^	<0.001
Extra PV foci, n (%)	229 (12.3)	78 (12.3)	87 (12.7)	64 (11.9)	0.913
Early recurrence, n (%)	808 (29.7)	231 (24.3)^a^	296 (29.4)^b^	281 (36.6)^c^	<0.001
Clinical recurrence, n (%)	874 (32.1)	232 (24.4)	317 (31.5)	325 (42.4)	<0.001
1-year recurrence, n (%)	419 (19.3)	109 (14.5)^a^	165 (20.5)^b^	145 (23.7)^c^	<0.001
1–3 years recurrence, n (%)	285 (16.6)	79 (12.5)^a^	85 (13.6)^a^	121 (26.2)^b^	<0.001
>3 years recurrence, n (%)	170 (20.0)	44 (13.9)^a^	67 (20.6)^b^	59 (28.2)^c^	<0.001
*P* value	0.046	0.652	0.001	0.360	
Genetic study (*n* = 2,051) (1)					
rs12646447*	1089 (50.6)	393 (53.0)	391 (49.5)	305 (49.2)	0.268

Values are presented as median (Q1–Q3 quartiles [25th and 75th percentiles]) or number (%).

*2,051 out of 2,913 patients had data on genetic study, and the presence of PV/LA%vol associated SNP was analyzed in 2,051 patients.

^a–c^There were significant differences between the groups with different alphabets.

AF, atrial fibrillation; CT, computed tomography; E/Em, ratio of the peak mitral flow velocity of the early rapid filling to the early diastolic velocity of the mitral annulus; LA, left atrium; LV, left ventricular; PV, pulmonary vein; PV/LA%vol, pulmonary vein to left atrium volume percent ratio; TIA, transient ischemic attack.

In Table 2, we divided the patients into three groups according to the PV/LA%vol tertiles. Although the pattern of clinical characteristics in T1–T3 was the same as that of LAD, the direction of each variable difference was opposite. On linear regression analysis (Model 1 in Table 3), PV/LA%vol was independently associated with lesser remodeled AF: paroxysmal AF (β = 0.41 [0.28–0.55], *p* < 0.001), male sex (β = 0.61 [0.45–0.76], *p* < 0.001), younger age (β = −0.01 [−0.02–0], *p* = 0.005), absence of hypertension (β = −0.22 [−0.35 – −0.09], *p* = 0.001), left ventricular ejection fraction (β = 0.01 [0–0.02], *p* = 0.030), and lower E/Em (β = −0.02 [−0.04 – −0.01], *p* = 0.007).

**TABLE 2:** Clinical and procedural characteristics among the three groups according to PV/LA%vol tertiles.

	T1 PV/LA%vol (<3.10) (*n* = 971)	T2 PV/LA%vol (≥3.10, <4.35) (*n* = 971)	T3 PV/LA%vol (≥4.35) (*n* = 971)	*P*
Age (year)	60.0 (53.0–67.0)^a^	60.0 (52.0–67.0)	59.0 (51.0–66.0)^b^	0.001
Male sex, n (%)	612 (63.0)^a^	727 (74.9)^b^	801 (82.5)^c^	<0.001
Paroxysmal AF, n (%)	505 (52.0)^a^	602 (62.0)^b^	658 (67.8)^c^	<0.001
AF duration (months)	24.0 (10.0–52.0)^a^	20.0 (7.5–48.0)	18.0 (8.0–36.0)^b^	0.010
Comorbidity, n (%)				
Hypertension	486 (50.1)^a^	488 (50.3)^a^	395 (40.7)^b^	<0.001
Diabetes mellitus	131 (13.5)	164 (16.9)	143 (14.7)	0.105
Stroke/TIA	117 (12.0)	112 (11.5)	91 (9.4)	0.135
Vascular disease	111 (11.4)^a^	108 (11.1)^a^	56 (5.8)^b^	<0.001
Heart failure	143 (14.7)	117 (12.0)	122 (12.6)	0.179
Body mass index (kg/m^2^)	24.8 (23.1–26.7)	24.8 (23.0–26.8)	24.7 (23.0–26.7)	0.668
CHA_2_DS_2_-VASc score	2.0 (1.0–3.0)^a^	1.0 (1.0–3.0)^a^	1.0 (0–2.0)^b^	<0.001
Echocardiography parameters				
LA dimension (mm)	43.0 (39.0–46.0)^a^	41.0 (37.0–45.0)^b^	39.0 (35.0–44.0)^c^	<0.001
LV ejection fraction (%)	64.0 (59.0–69.0)	64.0 (60.0–68.0)	64.0 (60.0–69.0)	0.198
E/Em	10.0 (8.0–12.4)^a^	9.0 (7.1–11.9)^b^	9.0 (7.1–11.0)^b^	<0.001
CT parameters				
LA volume (cm^3^)	165.6 (138.6–194.8)^a^	144.4 (122.2–173.3)^b^	131.4 (110.4–158.2)^c^	<0.001
PV volume (cm^3^)	3.9 (3.3–4.8)^a^	5.3 (4.4–6.3)^b^	7.6 (6.1–9.7)^c^	<0.001
PV/LA%vol (%)	2.5 (2.1–2.8)^a^	3.7 (3.4–4.0)^b^	5.5 (4.8–6.6)^c^	<0.001
PV anatomy, n (%)				<0.001
Normal PV anatomy	816 (84.0)^a^	884 (91.0)	925 (95.3)^b^	
Common PV trunk	43 (4.4)^a^	28 (2.9)	12 (1.2)^b^	
Accessory PV	112 (11.5)^a^	59 (6.1)	34 (3.5)^b^	
Ablation time (min)	74.3 (54.9–93.8)^a^	68.2 (45.1–88.5)^b^	43.2 (28.2–67.3)^c^	<0.001
Procedure time (min)	173.0 (140.0–209.0)^a^	162.0 (125.0–198.0)^b^	120.0 (88.0–160.0)^c^	<0.001
Ablation lesion, n (%)				
CPVI	971 (100)	971 (100)	971 (100)	1
Empirical extra-PV LA ablation	327 (33.7)^a^	246 (25.4)^b^	121 (12.5)^c^	<0.001
Mean LA voltage (mV) (*n* = 1,918)	1.1 (0.7–1.6)^a^	1.4 (0.9–1.9)^b^	1.7 (1.3–2.2)^c^	<0.001
Extra-PV foci, n (%)	86 (13.8)	73 (11.7)	70 (11.5)	0.395
Early recurrence, n (%)	342 (35.4)^a^	264 (27.8)^b^	202 (24.9)^c^	<0.001
Clinical recurrence, n (%)	407 (42.1)^a^	309 (32.6)^b^	158 (19.5)^c^	<0.001
1-year recurrence, n (%)	167 (19.0)	151 (18.5)	101 (21.5)	0.381
1–3 years recurrence, n (%)	153 (21.7)^a^	94 (14.3)^b^	38 (10.8)^c^	<0.001
>3 years recurrence, n (%)	87 (23.6)^a^	64 (18.6)^b^	19 (13.8)^c^	0.032
*P* value	0.142	0.070	<0.001	
Genetic study (*n* = 2,051)*				
rs12646447 (1)	329 (47.6)	360 (50.6)	400 (53.4)	0.090

Values are presented as median (Q1–Q3 quartiles [25th and 75th percentiles]) or number (%).

*2,051 out of 2,913 patients had data on genetic study, and the presence of PV/LA%vol associated SNP was analyzed in 2051 patients.

^a–c^There were significant differences between the groups with different alphabets.

AF, atrial fibrillation; CPVI, circumferential pulmonary vein isolation; E/Em, ratio of the peak mitral flow velocity of the early rapid filling to the early diastolic velocity of the mitral annulus; LA, left atrium; LV, left ventricular; PV, pulmonary vein; PV/LA%vol, pulmonary vein to left atrium volume percent ratio; T1, first tertile; T2, second tertile; T3, third tertile; TIA, transient ischemic attack.

**TABLE 3:** Linear regression analysis for PV/LA%vol.

	Univariate		Multivariate model 1		Multivariate model 2*	
	β (95% CI)	*P*	β (95% CI)	*P*	β (95% CI)	*P*
Paroxysmal AF	0.47 (0.34–0.60)	<0.001	0.41 (0.28–0.55)	<0.001	0.36 (0.20–0.51)	<0.001
Male sex	0.17 (0.54–0.83)	<0.001	0.61 (0.45–0.76)	<0.001	0.58 (0.40–0.76)	<0.001
Age	−0.11 (−0.02 – −0.01)	<0.001	−0.01 (−0.02–0)	0.005	−0.01 (−0.02 – −0)	<0.001
Body mass index	0 (−0.02–0.02)	0.830				
Hypertension	−0.10 (−0.47 – −0.21)	<0.001	−0.22 (−0.35 – −0.09)	0.001	−0.18 (−0.33 – −0.03)	0.023
Diabetes	−0.01 (−0.22–0.14)	0.632				
Heart failure	−0.03 (−0.35–0.03)	0.105				
LV ejection fraction	0.05 (0–0.02)	0.006	0.01 (0–0.02)	0.030	0.01 (0–0.02)	0.233
E/Em	−0.13 (−0.07 – −0.04)	<0.001	−0.02 (−0.04 – −0.01)	0.007	−0.02 (−0.04–0)	0.061
Extra-PV foci	−0.03 (−0.38–0.11)	0.270				
Genetic study (*n* = 2,051) *						
rs12646447	0.15 (0–0.30)	0.047			0.20 (0.05–0.35)	0.010

*In model 2, 2,051 out of 2,913 patients who had data on genetic study were analyzed, and the presence of PV/LA%vol associated SNP was adjusted with clinically significant variables in the univariate analysis.

AF, atrial fibrillation; CI, confidence interval; E/Em, ratio of the peak mitral flow velocity of the early rapid filling to the early diastolic velocity of the mitral annulus; LA, left atrium; LV, left ventricular; PV, pulmonary vein; PV/LA%vol, pulmonary vein to left atrium volume percent ratio.

### Atrial fibrillation recurrence after atrial fibrillation catheter ablation according to the PV/LA%vol

**FIGURE 1:**
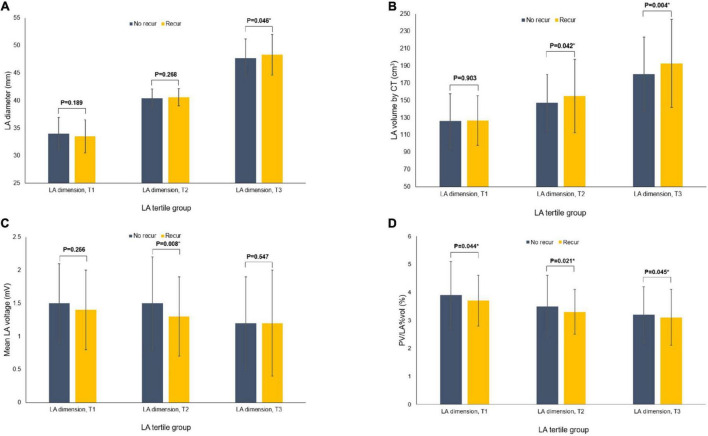
Comparisons of the variables according to the recurrence of AF at 1-year after the AFCA among the tertile groups of LA dimension. Comparisons of the LA dimension **(A)**, CT-measured LA volume **(B)**, mean LA voltage **(C)**, and PV/LA%vol **(D)**. AF, atrial fibrillation; AFCA, atrial fibrillation catheter ablation; CT, computed tomography; LA, left atrium or left atrial; PV, pulmonary vein; PV/LA%vol, pulmonary vein to left atrium volume percent ratio.

During 20.0 (8.0–45.0) months of follow-up, although the post-AFCA recurrence rate was higher in the higher tertile LAD group (*p* < 0.001, Table 1), LAD revealed significant difference according to the 1-year AF recurrence in the T3 group (*p* = 0.046) but not in T1 and T2 groups (Figure 1A). In patients who experienced AF recurrence within a year, LA volume measured by CT was higher in the T2 (*p* = 0.042) and T3 (*p* = 0.004, Figure 1B) groups, and LA voltage was lower in the T2 LAD group alone (*p* = 0.008, Figure 1C). However, PV/LA%vol was consistently lower in patients with 1-year AF recurrence in all groups (T1, *p* = 0.044; T2, *p* = 0.021; and T3, *p* = 0.045, Figure 1D).

**FIGURE 2:**
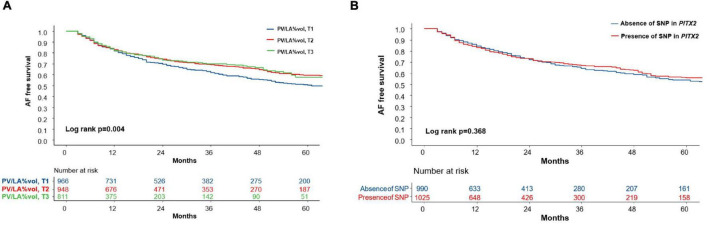
The Kaplan-Meier curve for clinical recurrence of AF according to the tertile groups of PV/LA%vol and presence of the *PITX2* gene. **(A)** The AF recurrence rate was significantly higher in the lowest tertile PV/LA%vol group (Log-rank *p* = 0.004). **(B)** However, AF recurrence did not differ according to the presence of an SNP in the *PITX2* gene (Log-rank *p* = 0.368). AF, atrial fibrillation; PV/LA%vol, pulmonary vein to left atrium volume percent ratio; SNP, single nucleotide polymorphism.

Atrial fibrillation recurrence rate was significantly higher in the lowest tertile PV/LA%vol group (Log-rank *p* = 0.004, Figure 2A). In the multivariate Cox regression analysis, PV/LA%vol (HR 0.91 [0.84–1.00], *p* = 0.044), persistent AF (HR 1.42 [1.12–1.80], *p* = 0.003), mean LA voltage (HR 0.84 [0.70–1.00], *p* = 0.049), extra-PV foci (HR 1.80 [1.38–2.34], *p* < 0.001), and LAD (Model 2; HR 1.03 [1.02–1.05], *p* < 0.001) were independently associated with clinical recurrence after AFCA (Table 4).

**TABLE 4:** Cox regression analysis for clinical recurrence after AFCA.

	Univariate		Multivariate model 1*		Multivariate model 2*	
	HR (95% CI)	*P*	HR (95% CI)	*P*	HR (95% CI)	*P*
Persistent AF	1.54 (1.35–1.76)	<0.001	1.42 (1.12–1.80)	0.003	1.35 (1.07–1.71)	0.013
Female	1.17 (1.01–1.35)	0.038	1.10 (0.88–1.37)	0.387	1.19 (0.95–1.48)	0.128
Age	1.00 (1.00–1.01)	0.583				
Body mass index	1.02 (1.00–1.04)	0.110				
Comorbidity						
Hypertension	1.12 (0.98–1.28)	0.103				
Diabetes	1.11 (0.93–1.33)	0.246				
Heart failure	1.26 (1.04–1.53)	0.017	1.15 (0.86–1.53)	0.353	1.01 (0.76–1.36)	0.926
Vascular disease	0.93 (0.76–1.15)	0.523				
LV ejection fraction	0.99 (0.98–1.00)	0.012	0.99 (0.98–1.01)	0.403	1.00 (0.98–1.01)	0.439
E/Em	1.01 (1.00–1.03)	0.084				
Mean LA voltage	0.64 (0.56–0.72)	<0.001	0.84 (0.70–1.00)	0.049	0.86 (0.72–1.02)	0.080
Extra-PV foci	1.99 (1.59–2.49)	<0.001	1.80 (1.38–2.34)	<0.001	1.82 (1.40–2.37)	<0.001
Empirical extra-PV LA ablation	1.54 (1.34–1.77)	<0.001	1.13 (0.89–1.42)	0.325	1.10 (0.87–1.39)	0.420
LA dimension*	1.04 (1.03–1.06)	<0.001			1.03 (1.02–1.05)	<0.001
PV/LA%vol*	0.89 (0.84–0.94)	<0.001	0.91 (0.84–1.00)	0.044		
PV anatomy	1.06 (0.95–1.18)	0.312				
Genetic study (*n* = 2,051) (2)						
rs12646447^†^	0.93 (0.80–1.09)	0.371				

*Two multivariate models were separately presented because LA dimension and PV/LA volume ratio had a multicollinearity to each other.

^†^Univariate analysis was performed in 2051 out of 2913 patients who had data on genetic study.

AF, atrial fibrillation; AFCA, atrial fibrillation catheter ablation; CI, confidence interval; E/Em, ratio of the peak mitral flow velocity of the early rapid filling to the early diastolic velocity of the mitral annulus; LA, left atrium; LV, left ventricular; PV, pulmonary vein; PV/LA%vol, pulmonary vein to left atrium volume percent ratio.

### PV/LA%vol as a predictor of atrial fibrillation recurrence in patients without significant left atrial enlargement

**FIGURE 3:**
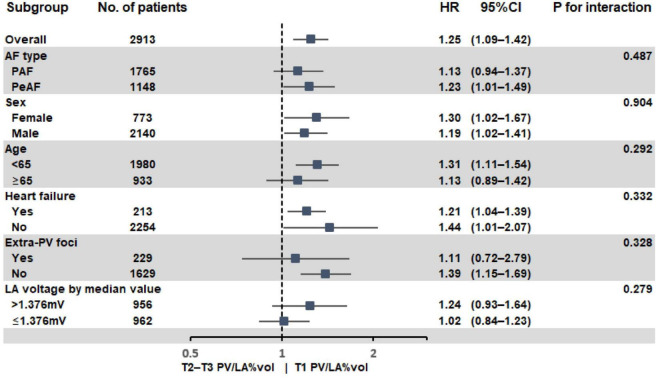
Subgroup analysis of AF recurrence after AFCA according to PV/LA%vol. AF, atrial fibrillation; AFCA, atrial fibrillation catheter ablation; LA, left atrium or left atrial; PV, pulmonary vein; PV/LA%vol, pulmonary vein to left atrium volume percent ratio.

**FIGURE 4:**
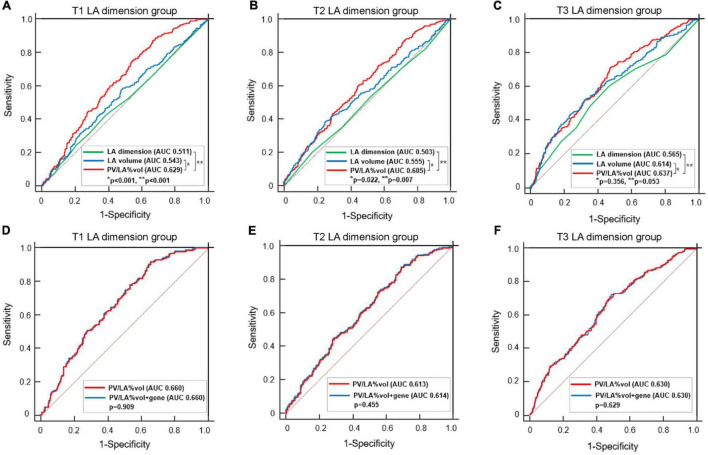
Comparisons of the predictive value of AF recurrence among LA dimension, LA volume, PV/LA%vol, and PV/LA%vol with *PITX2* genome in the tertile groups according to the LA dimension. Comparisons of AUCs between PV/LA%vol, LA volume, and LA dimension in the T1 **(A)**, T2 **(B)**, and T3 **(C)** LA dimension groups. Comparisons of AUCs between PV/LA%vol and PV/LA%vol with *PITX2* gene in the T1 **(D)**, T2 **(E)**, and T3 **(F)** LA dimension groups. AF, atrial fibrillation; AUC, Area under the receiver operating characteristic curve; LA, left atrium or left atrial; PV/LA%vol, pulmonary vein to left atrium volume percent ratio.

In the subgroup analyses, T1 PV/LA%vol was independently associated with post-AFCA recurrence regardless of sex or the presence of heart failure (Figure 3). T1 PV/LA%vol was also independently associated with AF recurrence in patients with persistent AF, those older than 65 years, or those with no extra-PV foci without intergroup differences (Figure 3). When we compared the overall sensitivity and specificity of LAD, LA volume, and PV/LA%vol for clinical recurrence, the AUC of PV/LA%vol was better than that of LAD in T1 (AUC 0.629 vs. 0.511, *p* < 0.001) or T2 (AUC 0.605 vs. 0.503, *p* = 0.007), but not in the T3 LAD group (AUC 0.637 vs. 0.565, *p* = 0.053, Figures 4A–C). Moreover, PV/LA%vol also showed higher AUC value than LA volume in T1 (AUC 0.629 vs. 0.543, *p* < 0.001) or T2 (AUC 0.605 vs. 0.555, *p* = 0.022), but not in the T3 LAD group (AUC 0.637 vs. 0.614, *p* = 0.356, Figures 4A–C).

In sensitivity analysis with selected patients who underwent circumferential PV isolation alone, PV/LA%vol (HR 0.90 [0.82–1.00], *p* = 0.040) was independently associated with AF recurrence after adjustment with other variables (Supplementary Table 1). Furthermore, AUC of PV/LA%vol was also significantly better than that of LAD in T1 (AUC 0.651 vs. 0.512, *p* < 0.001) and T2 (AUC 0.593 vs. 0.507, *p* = 0.008) LAD groups in selected patients (Supplementary Figures 2A–C).

### Genetic influence on PV/LA%vol

We divided the 2,151 patients into two cohorts randomly and found eight *PITX2* SNPs in cohort 1 and four *PITX2* SNPs in cohort 2 that were associated with PV/LA%vol by linear regression analysis. Among them, one *PITX2* SNP, rs12646447, was replicated to be associated with PV/LA%vol in two independent cohorts (Supplementary Table 2). The prevalence of rs12646447 did not differ among the T1–T3 PV/LA%vol groups (*p* = 0.090, Table 2). On multivariate linear regression analyses, *PITX2* rs12646447 was independently associated with PV/LA%vol (β = 0.20 [0.05–0.35], *p* = 0.010, Model 2 in Table 3), but not with the AF recurrence after AFCA (Lon-rank *p* = 0.368, Figure 2B; HR 0.93 [0.80–1.09], *p* = 0.371, Table 4). Although the *PITX2* genome was associated with PV/LA%vol, it did not have incremental benefits in addition to PV/LA%vol as a predictor of AF recurrence after AFCA (Figures 4D–F).

## Discussion

### Main findings

This study explored the atrial structural factors affecting post-ablation AF recurrence in patients without significant LA enlargement. We found that PV/LA%vol measured on cardiac CT imaging was consistently predictive of 1-year AF recurrence in all tertile groups classified by LAD and was independently associated with overall recurrence after AFCA. PV/LA%vol had a significantly better predictive power for AF recurrence than LAD in patients with normal or mild LA enlargement (T1 and T2 LAD groups). We also confirmed the association between PV/LA%vol and the *PITX2* gene, which plays a role in LA-PV development. It seems that both acquired atrial remodeling and innate genetic factors influence PV/LA%vol. However, *PITX2* genetic factors did not affect the long-term rhythm outcomes after AFCA, which appeared to properly control its genetic influence.

### Left atrial dimension vs. left atrial and pulmonary vein volume in atrial fibrillation

Dilatation of the LA reflects AF remodeling or progression, and is known to be related to the risk of recurrence after AFCA (13, 14). Because it is easy to obtain, LAD measured on echocardiography has been widely used to predict AF recurrence after AFCA (3). However, because the LA is an asymmetrical cavity, the LAD could be insensitive to changes in its size. Previous studies have reported that LA volume measured by MDCT is a good predictive marker of an AF recurrence after AFCA (15). Several efforts have been made to relate PV morphology with arrhythmogenicity, but the outcomes have been inconsistent (8, 9, 16). A large PV size was recently postulated as being associated with the risk of AF recurrence (17, 18). We also found that the patients with larger LAD have higher PV volume and lower PV/LA%vol in this study (Table 1).

### PV/LA%vol and clinical recurrence after atrial fibrillation catheter ablation

Although LAD or volume has been traditionally used as a predictor of recurrence after AFCA (3, 19), AF recurrence still occurs even in patients with normal LA size or without any significant atrial remodeling (20). Therefore, LAD may not be suitable for predicting AF recurrence after AFCA in patients without enlarged LA. Recent studies reported that large PV volume on CT images is associated with arrhythmogenic PV trigger or AF recurrence after catheter ablation (9, 17, 18). In the present study, we investigated a new parameter, PV/LA%vol, and found that a smaller PV/LA%vol was related to a poor rhythm outcome after AFCA. There are several potential mechanisms underlying this result. First, a relatively high wall stress acts on LA as compared to PVs in a low PV/LA%vol condition and is associated with a high recurrence rate (21). Second, patients with a low PV/LA%vol had a low LA voltage and high E/Em (Table 2), which are factors that are related to post-ablation recurrence (6, 22, 23). Third, PV/LA%vol is affected by innate genetic factors such as *PITX2* gene as well as acquired AF progression and atrial remodeling. Although *PITX2* gene did not directly affect the recurrence rate in this study, it affected AAD responsiveness (24), and morphological PV-LA development may play some role as AF recurrence mechanism in patients with mild LA enlargement. According to the outcomes of the present study, the new parameter, PV/LA%vol, provided additional information about the procedural effect prior to AFCA.

### Genetic association of *PITX2* gene and PV/LA%vol

Atrial fibrillation is a heritable disease, and *PITX2* gene is known as the most common AF-associated genome among over 100 related SNPs (10, 25). *PITX2* gene is embryologically involved in LA and right atrium asymmetry, PV, and pacemaker cell development and is electrophysiologically related to PV-triggered activity (10, 11, 25–28). Clinically, AAD responsiveness or recurrence after cardioversion differs among *PITX2* variants in patients with AF (24); however, its relationship with the rhythm outcome after AFCA is controversial (28, 29). We found here for the first time the association between *PITX2* rs12646447 and PV/LA%vol, which was a predictor of post-AFCA recurrence in patients with minimal or mild atrial remodeling. The rs12646447 is also associated with cardioembolic stroke, and the risk-allelic frequency was 0.49 in the Asian population (30). Nevertheless, this genetic predisposition for PV/LA%vol was not significantly associated with AF recurrence following catheter ablation, which is consistent with the findings of our previous study (28). That suggested that PV isolation effectively controls the *PITX2* genetic influence on the mechanism of AF.

### Study limitations

This study had several limitations. First, the study may have included a highly selected group of patients referred for AFCA due to its single-center prospective observational nature. However, we kept a consistent ablation protocol by obtaining the data from a single center. In addition, the mean CHA_2_DS_2_-VASc score of the patients included in this study was relatively low as in another AFCA studies. Therefore, we cannot generalize the results of this study to all AF population. Second, although we kept a consistent ablation protocol used by experienced operators, the catheter technology and mapping technologies kept changing during the long enrollment period. Third, there is no gold standard for the boundary of the PV and LA for volume measurements, and PV anatomy is variable. Therefore, in this study, a single technician, who was blinded to the clinical factors, measured the PV/LA%vol using a consistent technique. Fourth, we evaluated the susceptible SNPs of the *PITX2* gene screened using a commercial GWAS kit. SNPs associated with PV/LA%vol might have been missing. Moreover, our study included a highly selected group of Korean patients. Therefore, the generalizability of our results requires testing in further large-scale multicenter studies.

## Conclusion

After the adjustment for LA volume, a smaller PV volume (PV/LA%vol) was independently associated with poorer rhythm outcomes after AFCA. Although there was genetic association between *PITX2* gene and PV/LA%vol, no significant genetic predisposition was seen with the rhythm outcome after catheter ablation for AF.

## Data Availability Statement

The original contributions presented in the study are included in the article/Supplementary material, further inquiries can be directed to the corresponding author.

## Ethics statement

The studies involving human participants were reviewed and approved by the Institutional Review Board of the Yonsei University Health System. The patients/participants provided their written informed consent to participate in this study.

## Author contributions

J-HL and H-NP participated in designing of the work and statistical analysis, and drafted the manuscript. IH conducted the genetic analysis. HY, T-HK, J-SU, BJ, M-HL, and H-NP contributed to data collection. All authors were involved in interpretation of the results, read, and approved the manuscript before its submission.

## Conflict of Interest

The authors declare that the research was conducted in the absence of any commercial or financial relationships that could be construed as a potential conflict of interest.

## Publisher’s Note

All claims expressed in this article are solely those of the authors and do not necessarily represent those of their affiliated organizations, or those of the publisher, the editors and the reviewers. Any product that may be evaluated in this article, or claim that may be made by its manufacturer, is not guaranteed or endorsed by the publisher.
