# Supplementary material for: Lower pulmonary vein-to-left atrium volume ratio predicts poor rhythm outcome after atrial fibrillation catheter ablation
Source: Front Cardiovasc Med. 2022 Jul 15;9:934168. doi: 10.3389/fcvm.2022.934168 (PMC9334901; doi:10.3389/fcvm.2022.934168)
Supplement: Supplementary file 1 [file Data_Sheet_1.pdf]

Supplementary Material

Supplementary Figure 1. Representative three-dimensional reconstructed LA images and boundary of LA and PV volume.

Supplementary Figure 2. Comparisons of the predictive value of AF recurrence between LA dimension and PV/LA%vol in the tertile groups according to the LA dimension in selected patients who underwent circumferential PV isolation alone.

Supplementary Table 1. Cox regression analysis for clinical recurrence after AFCA in selected patients who underwent PV isolation alone.

Supplementary Table 2. Associated SNPs in *PITX2* with PV/LA%vol in two different cohorts.

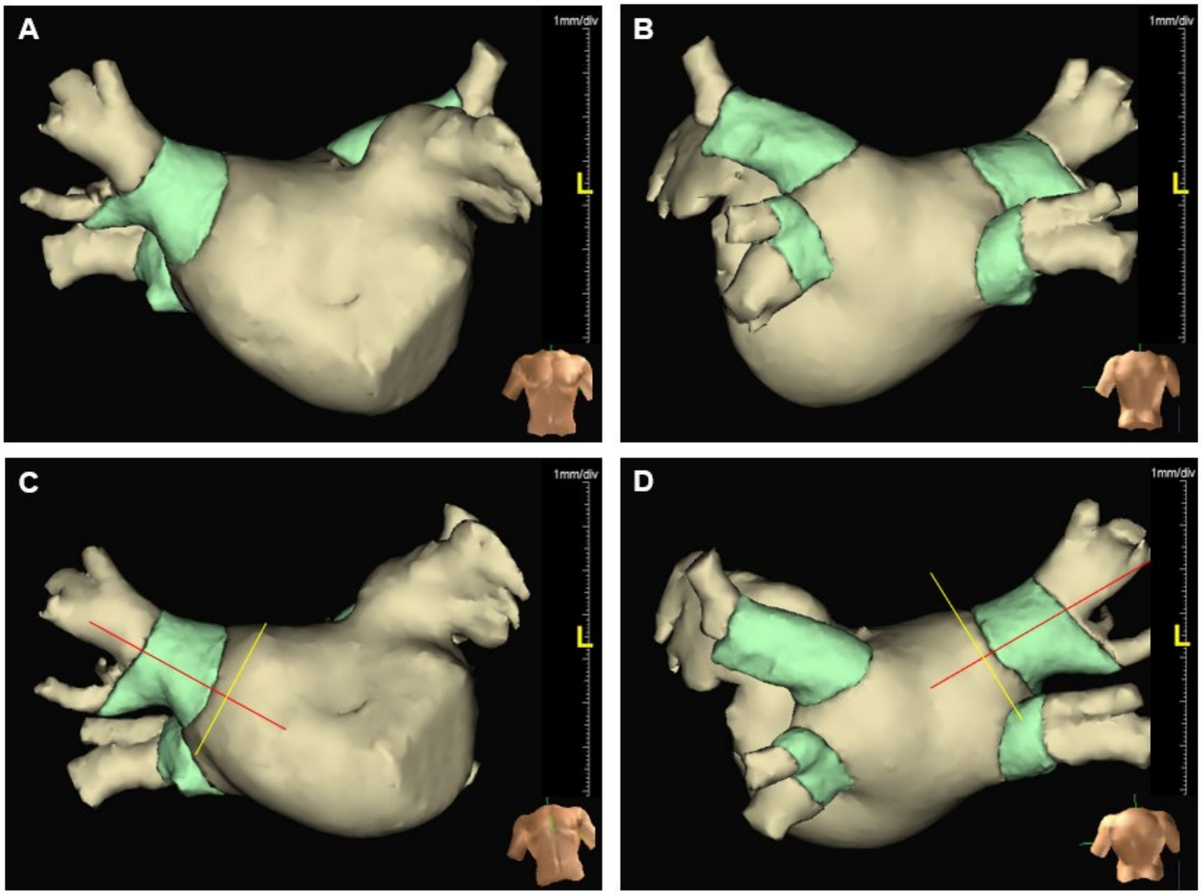

Supplementary Figure 1. Representative three-dimensional reconstructed LA images and boundary of LA and PV volume.

Three-dimensional reconstructed LA images in the AP (A) and PA views (B). The boundary of the PV was divided from the PV ostia to the primary tributaries with equal horizontal extension bilaterally in the AP (C) and PA views (D).

AP, anteroposterior; LA, left atrium or left atrial; PA, posteroanterior; PV, pulmonary vein.

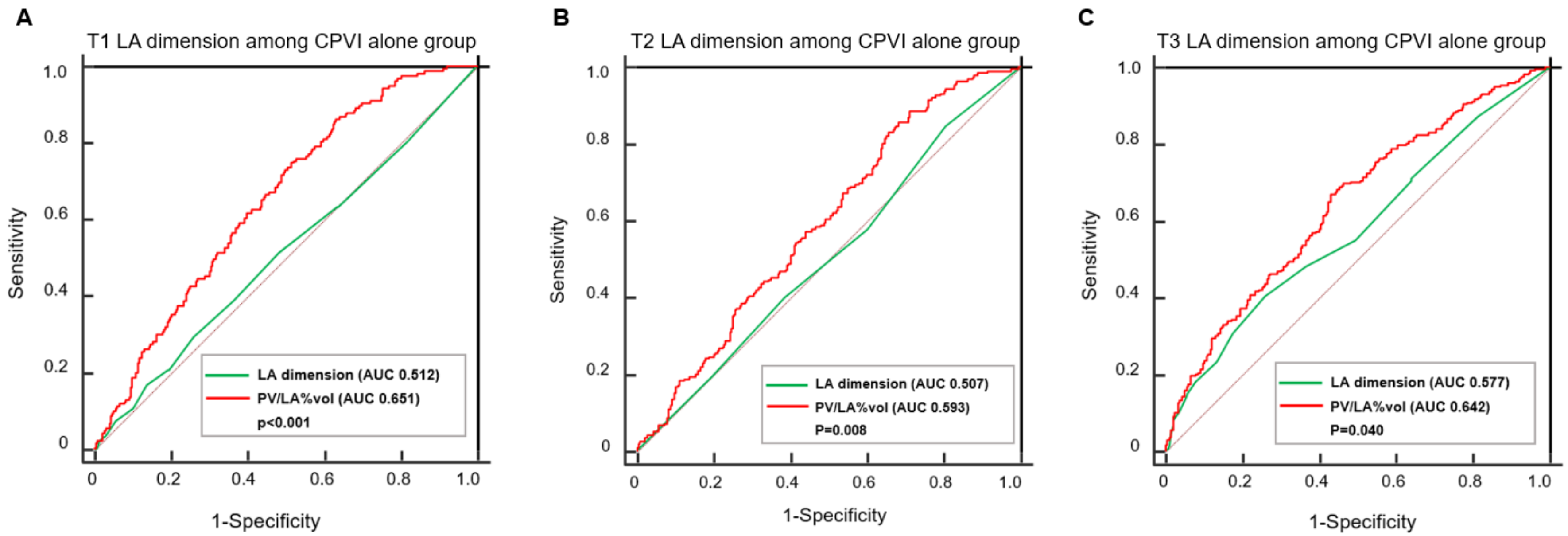

Supplementary Figure 2. Comparisons of the predictive value of AF recurrence between LA dimension and PV/LA%vol in the tertile groups according to the LA dimension in selected patients who underwent circumferential PV isolation alone.

Comparisons of AUCs between PV/LA%vol and LA dimension in the T1 (A), T2 (B), and T3 (C) LA dimension groups.

AF, atrial fibrillation; AUC, Area under the receiver operating characteristic curve; CPVI, circumferential pulmonary vein isolation; LA, left atrium or left atrial; PV/LA%vol, pulmonary vein to left atrium volume percent ratio.

Supplementary Table 1. Cox regression analysis for clinical recurrence after AFCA in selected patients who underwent PV isolation alone.

	Univariate		Multivariate	
	HR (95% CI)	P	HR (95% CI)	P
Persistent AF	1.42 (1.18–1.72)	<0.001	1.52 (1.18–1.95)	0.001
Female	1.27 (1.06–1.51)	0.009	1.49 (1.17–1.90)	0.001
Age	1.00 (0.99–1.01)	0.837		
Body mass index	1.02 (0.99–1.04)	0.219		
Comorbidity				
Hypertension	1.15 (0.98–1.36)	0.088		
Diabetes	1.15 (0.92–1.44)	0.212		
Heart failure	1.33 (1.02–1.72)	0.033	1.28 (0.87–1.89)	0.206
Vascular disease	0.75 (0.57–1.01)	0.054		
LV ejection fraction	0.99 (0.98–1.00)	0.029	1.00 (0.98–1.01)	0.765
E/Em	1.02 (1.00–1.04)	0.076		
Extra-PV foci	2.35 (1.74–3.17)	<0.001	2.22 (1.64–3.00)	<0.001
PV/LA%vol	0.90 (0.84–0.97)	0.003	0.90 (0.82–1.00)	0.040
Genetic study (n=1627)				
rs12646447†	0.96 (0.79–1.16)	0.655		

† Univariate analysis was performed in 1627 out of 2219 patients who had data on genetic study.

AF, atrial fibrillation; AFCA, atrial fibrillation catheter ablation; CI, confidence interval; E/Em, ratio of the peak mitral flow velocity of the early rapid filling to the early diastolic velocity of the mitral annulus; LA, left atrium; LV, left ventricular; PV, pulmonary vein; PV/LA%vol, pulmonary vein to left atrium volume percent ratio.

Supplementary Table 2. Associated SNPs in *PITX2* with PV/LA%vol in two different cohorts.

Chr	SNP	Position	Closest gene	Cohort 1 (n=1020)			Cohort 2 (n=1131)		
				β	95% CI	P	β	95% CI	P
**4**	**rs12646447**	**110778170**	***PITX2***	**0.12**	**0.02–0.23**	**0.020**	**0.17**	**0–0.33**	**0.045**
4	rs10019689	110728713	*PITX2*	0.12	0.02–0.22	0.017	NA	NA	NA
4	rs17042171	110787131	*PITX2*	0.12	0.02–0.23	0.020	NA	NA	NA
4	rs2200733	110789013	*PITX2*	0.12	0.02–0.22	0.022	NA	NA	NA
4	rs72672233	111070319	*PITX2*	0.11	0–0.23	0.048	NA	NA	NA
4	rs297014	111082215	*PITX2*	-0.11	-0.2–-0.02	0.022	NA	NA	NA
4	rs145701953	111416401	*PITX2*	-0.32	-0.64–0	0.048	NA	NA	NA
4	rs13131353	112878619	*PITX2*	-0.15	-0.20–-0.02	0.020	NA	NA	NA
4	rs10033464	110799605	*PITX2*	NA	NA	NA	0.22	0.01–0.43	0.041
4	rs13130446	110818000	*PITX2*	NA	NA	NA	0.24	0.08–0.39	0.003
4	rs11098092	110877045	*PITX2*	NA	NA	NA	0.18	0.03–0.34	0.020

NA, not available; PV/LA%vol, pulmonary vein to left atrium volume percent ratio; SNP, single nucleotide polymorphism.
